# Health Risk Assessment of Heavy Metals in Groundwater of Hainan Island Using the Monte Carlo Simulation Coupled with the APCS/MLR Model

**DOI:** 10.3390/ijerph19137827

**Published:** 2022-06-26

**Authors:** Huanhuan Shi, Min Zeng, Hongxia Peng, Changsheng Huang, Huimin Sun, Qingqin Hou, Pengcheng Pi

**Affiliations:** 1 School of Environmental Studies, China University of Geosciences, Wuhan 430078, China; shihh@cug.edu.cn; 2 School of Geography and Information Engineering, China University of Geosciences, Wuhan 430078, China; sun2544154846@163.com (H.S.); 17603907029@163.com (Q.H.); ppc0428@126.com (P.P.); 3 Wuhan Center of Geological Survey of China Geological Survey, Wuhan 430205, China; huangzhangsheng@mail.cgs.gov.cn

**Keywords:** heavy metals in groundwater, APCS/MLR model, source apportionment, Monte Carlo simulation, health risk

## Abstract

Groundwater is a significant component of water resources, but drinking groundwater with excessive heavy metals (HMs) is harmful to human health. Currently, quantitative source apportionment and probabilistic health risk assessment of HMs in groundwater are relatively limited. In this study, 60 groundwater samples containing seven HMs were collected from Hainan Island and analyzed by the coupled absolute principal component scores/multiple linear regression (APCS/MLR), the health risk assessment (HRA) and the Monte Carlo simulation (MCS) to quantify the pollution sources of HMs and the health risks. The results show that the high-pollution-value areas of HMs are mainly located in the industry-oriented western region, but the pollution level by HMs in the groundwater in the study area is generally low. The main sources of HMs in the groundwater are found to be the mixed sources of agricultural activities and traffic emissions (39.16%), industrial activities (25.57%) and natural sources (35.27%). Although the non-carcinogenic risks for adults and children are negligible, the carcinogenic risks are at a high level. Through analyzing the relationship between HMs, pollution sources, and health risks, natural sources contribute the most to the health risks, and Cr is determined as the priority control HM. This study emphasizes the importance of quantitative evaluation of the HM pollution sources and probabilistic health risk assessment, which provides an essential basis for water pollution prevention and control in Hainan Island.

## 1. Introduction

Groundwater is a widespread yet fragile water resource, bearing the essential functions of water resource guarantee and ecological maintenance. In recent years, with the increase in population and the intensification of resource consumption, groundwater resources are facing increasing degrees of pressure [1,2]. For example, various pollutants such as heavy metals (HMs), nitrates, chloride ions, and polycyclic aromatic hydrocarbons have entered groundwater due to the impact of traffic discharge, agricultural cultivation, industrial production, and seawater intrusion, which have seriously deteriorated the groundwater quality [3,4]. HMs are persistent, non-degradable and highly toxic pollutants [5,6,7] and can enter the human body through the food chain, and eventually pose some negative influence on human health. Previous research has shown that the excessive ingestion of Cr and Cd all severely affect the normal functions of the nervous system, liver and kidney [8,9]. As for Pb, it partly explains the origins of symptoms such as memory loss, anemia, mental retardation, lassitude, and abdominal discomfort [10]. Furthermore, a high concentration of Fe promotes bacterial growth in water and causes an objectionable odor in the water system [11]. Given such potential threats from these HMs, understanding the pollution levels, sources, and health risks of HMs in groundwater is critical for human health and ecological safety.

Numerous existing studies of HMs have focused on pollution level investigation, risk assessment and remediation strategy provision, but little attention has been paid to identifying and quantifying pollution source apportionment [12,13]. Previously, the pollution sources of HMs have been mainly identified by qualitative identification, and multivariate statistical methods such as the principal component analysis (PCA), factor analysis (FA), and correlation analysis (CA) are mainly applied to categorize the HMs in a downscaled manner [14,15]. However, these methods cannot quantify the contribution of different pollution sources, resulting in a lack of appropriate targeting of the pollution prevention efforts. In recent years, the absolute principal component score–multiple linear regression (APCS/MLR) method has been increasingly applied to quantify the source identification of HMs [16,17]. APCS/MLR is a reverse traceability method, which does not require the construction of the contaminant source composition profiles and can quantitatively calculate the contributions (in %) of different pollution sources to each HM, and thus helps determine the key pollution sources [18]. Studies of the quantitative source resolution of HMs using the APCS/MLR method have focused on media such as soil, sediment, and dust [17,19,20], whereas studies on groundwater are relatively lacking. In this study, the APCS/MLR model was used to quantify the sources of HMs in groundwater to obtain more accurate and reliable results.

Traditional health risk assessment (HRA) is mainly a deterministic assessment, mostly using theoretical maximum parameters to calculate the risk values [21]. However, the parameters of exposure assessment and risk characterization usually have uncertainties, which lead to some errors in the assessment results [22,23]. Probabilistic methods can reduce the estimation bias caused by uncertainty and obtain more accurate evaluation results [24]. The Monte Carlo simulation (MCS) is an effective probabilistic evaluation method that not only quantifies the uncertainty in the risk assessment but also identifies the degree of influence of the exposure pathways and parameters on the risk, and has been increasingly applied to the risk assessment of HMs [25,26]. Therefore, we attempt to take advantage of this by using a combination of the HRA and the MCS to assess the health risk in this study.

So far, the contribution of pollution sources to the health risk by HMs has been largely ignored [27]. To further investigate the contribution of different pollution sources to the health risk, this study quantifies the health risk of HMs from different pollution sources in groundwater based on the coupling of the APCS/MLR model and the HRA model. In addition, previous studies on the health risk of HMs in groundwater have mainly focused on the regional scale such as rural, mining, agricultural areas, and cities [28,29], whereas related studies on larger scale islands were limited. Hainan Island is China’s experimental zone of reform and opening-up, with a unique geographical location and rich natural resources. In recent years, with the strong support of national policies, industries such as tourism, high technology and cold chain logistics have developed rapidly on the island, inevitably putting pressure on the ecological environment. However, little attention has been paid to the environmental assessment of Hainan Island yet; in particular, it still lacks corresponding research on the health risk assessment of HMs in groundwater.

This study quantifies the health risks of different pollution sources of the groundwater on a large scale in Hainan Island based on the coupling of the APCS/MLR model, the HRA and the MCS. The main objectives are to (1) evaluate the pollution levels of HMs in the groundwater, (2) quantify the potential pollution sources of HMs, (3) assess the health risks of HMs in the groundwater from different pollution sources, and (4) determine the priority HMs for risk control by analyzing the relationships among the pollution levels of the HMs, the potential pollution sources, and the health risks. Overall, this study can provide a scientific reference for pollution control of the groundwater and the health risks to the residents in Hainan Island.

## 2. Materials and Methods

### 2.1. Study Area

Hainan Island is the second largest island in China, located in the northwestern part of Hainan Province, with geographical coordinates between 108°37′ and 111°03′ E and 18°10′ and 20°10′ N. The total area is about 3.4 × 10^4^ km^2^. Hainan Island has a tropical monsoon climate, with an average annual temperature of 22–26 °C. Rainfall is mainly concentrated from May to July, with a distinction between the dry season and the rainy one. The groundwater in the area is mainly recharged by atmospheric rainfall, with some sections by surface water. The landform type is mainly mountainous and hilly in the study area, with low and flat surroundings and towering in the middle. The local rocks are mainly magmatic rocks and the soil type is mainly latosol. Cropland is the main land-use type in Hainan Island. The industry has been concentrated in the western region, and the key pillar industries are chemistry, medicine, paper making, electrical power and food processing. At the same time, Hainan Island is known for its tropical agricultural crop production in south China, such as the rich crops of rubber, coconut, betel nut, and pepper. In recent years, driven by the strong support of national policy, the industry, agriculture and tourism of Hainan Island have developed rapidly. Coupled with the impact of land reclamation, the decline of groundwater levels, water pollution and seawater intrusion have become the most urgent threats to the safety of groundwater resources in Hainan.

### 2.2. Sampling and Testing

**Figure 1:**
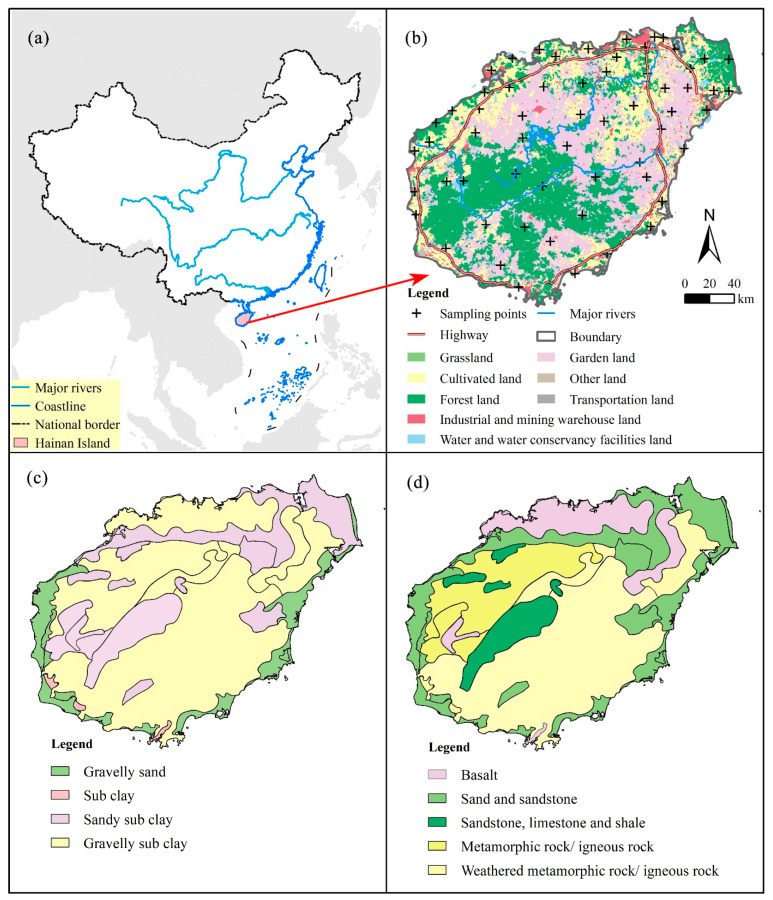
(**a**) The location of the study area in China; (**b**) the sampling sites’ distribution and land use classification; (**c**) the soil media and (**d**) the aquifer media in Hainan Island.

In this study, using GIS technology, Hainan Island was divided into 60 grids, each of which is 20 km × 20 km. The center of each grid is the preset sampling point, and a total of 60 samples were collected in January and February of 2015 (dry season) by adopting this grid sampling strategy. There are two reasons for sampling the groundwater in January and February. Firstly, the specifications for groundwater contamination investigation and evaluation (DD2008-01) recommend that the monitoring wells of background value and the regionally controlled pore confined water wells be sampled once a year during the dry period. Secondly, Hainan Island has high precipitation during the wet season, resulting in more interference to the groundwater. Therefore, the groundwater in the dry season can better represent the water quality of the study area. The locations of the groundwater sampling points are shown in Figure 1.

A HACH40d portable water quality analyzer was used on site to determine the basic water quality indexes such as water temperature, pH, conductivity, and dissolved oxygen, etc., of the samples. The collected water samples were filtered by a 0.45 μm aqueous filter membrane and loaded into 500 mL polyethylene bottles, with about 3 mL of 65% HNO_3_ added to adjust the pH value to below 2, and sealed and stored in a portable refrigerator at 4 °C. Then, these samples were sent to the Mineral Resources Supervision and Testing Center of Changsha of the Ministry of Land and Resources of China for testing. The concentrations of Cr, Mn, Fe, Cu, Zn, Cd and Pb were determined by an inductively coupled plasma mass spectrometer (ICPMS-7700X, Agilent Technologies, Tokyo, Japan) in the testing. For quality control, a calibration blank and an independent calibration verification standard were analyzed for every ten samples to confirm the calibration status of the ICP-MS. All blank test results were below the detection limit, and the blank spike recovery rate was between 95% and 120%.

### 2.3. Methods

#### 2.3.1. Water Quality Evaluation

Water quality index (WQI) can effectively convert the data of water quality characteristics and standard parameters into a single value representing the water quality, so as to simplify the complex data processing in evaluating the drinking water quality [30]. The calculation formulas are shown below [31]:

(1) $$\mathrm{Wi}=\frac{\mathrm{wi}}{\sum \mathrm{wi}}$$

(2) $$\mathrm{qi}=\frac{\mathrm{Ci}}{\mathrm{Si}}\times 100$$

(3) SIi=Wi×qi

(4) $$\mathrm{WQI}=\overset{N}{\underset{i=1}{\sum }}\mathrm{SIi}$$

where wi is the weight of each parameter; Wi the relative weight, Ci the measured concentration, mg/L, Si the grade III groundwater quality standard value (GB/T 14848-2017) [32], mg/L, qi the quality grade based on the concentration of the ^i^th parameter, and SIi the water quality sub index of the ^i^th parameter. According to the calculation results of WQI, the water quality can be divided into five categories: excellent (WQI < 50), good (50 ≤ WQI < 100), poor (100 ≤ WQI < 200), very poor (200 ≤ WQI ≤ 300) and unfit for drinking (WQI > 300) [31]. The relevant values are shown in Table S1.

The pollution evaluation index (PEI) is an effective measure to assess the level of HMs pollution and is used in this study to classify the water quality [33], which is calculated by Equation (5).

(5) $$\mathrm{PEI}=\overset{N}{\underset{i=1}{\sum }}\frac{\mathrm{Hc}}{\mathrm{Hmac}}$$

where Hc and Hmac are the measured value and maximum allowable concentration of each HM, μg/L, respectively. In this study, Hmac represents the grade III groundwater quality standard value (GB/T 14848-2017). The PEI results indicate the degree of pollution of low (HEI < 40), medium (40 ≤ HEI ≤ 80), or high (HEI > 80) [31].

#### 2.3.2. The APCS/MLR Model

The APCS/MLR model was used to obtain the absolute principal component factor scores based on factor analysis after standardizing the raw data and combining with the multiple linear regression model to calculate the contribution rate (%) of various sources of each HM [34]. The multiple linear regression calculation is shown in Equation (6) [27],

(6) $$C_{n}{=\xi }_{0}+\overset{p}{\underset{k-1}{\sum }}\xi_{k}{\times \mathrm{APCS}}_{k}$$

where C_n_ is the concentration of HM_n_, ξ_0_ a constant term (the intercept of the regression of HM_n_, ξ_k_ the regression coefficient, APCS_k_ the absolute principal component score of source p for the considered samples, ξ_k_ × APCS_k_ represents the contribution of source p to C_n_, and the value of ξ_k_ × APCS_k_ the average contribution of source p to C_n_.

#### 2.3.3. Health Risk Assessment

For HMs in the water environment, ingestion and dermal contact play the most important role [35]. In this study, the health risk of different populations (adult males, adult females and children) through ingestion and dermal contact of exposure pathways to HMs was assessed using the HRA model as recommended by the United States Environmental Protection Agency (USEPA). In addition, adults are considered as the general population and children as the sensitive group. Based on previous studies, seven HMs were classified as non-carcinogenic (Mn, Fe, Cu, and Zn) and carcinogenic (Cr, Cd, and Pb) elements [29,36], respectively.

The non-carcinogenic risks can be calculated using the hazard quotient (HQ). To assess the cumulative non-carcinogenic risk, the hazard index (HI) is introduced [22], which is the sum of the HQ for the two exposure routes. The relevant calculation Equations are as follows:

(7) $${\mathrm{ADD}}_{\mathrm{oral}}=\frac{C_{w}\times \mathrm{IR}\times \mathrm{EF}\times \mathrm{ED}}{\mathrm{BW}\times \mathrm{AT}}$$

(8) $${\mathrm{ADD}}_{\mathrm{dermal}}=\frac{C_{w}\times \mathrm{SA}\times \mathrm{PC}\times \mathrm{ET}\times \mathrm{EF}\times \mathrm{ED}\times \mathrm{CF}}{\mathrm{BW}\times \mathrm{AT}}$$

(9) $$\mathrm{HI}=\sum \mathrm{HQ}=\sum \frac{{\mathrm{ADD}}_{\mathrm{ij}}}{{\mathrm{RfD}}_{\mathrm{ij}}}$$

where ADD_oral_ and ADD_dermal_ are the average daily exposure dose through ingestion and dermal contact, respectively. For HI > 1, it indicates severe non-carcinogenic risk on human health, whereas HI < 1 is considered to be safe.

The carcinogenic risk can be calculated using the incremental lifetime cancer risk (ILCR), and the total cancer risk (TCR) represents the sum of the potential carcinogenic risks. The relevant calculation y Equations are as follows [28]:

(10) $$\mathrm{ILCR}=\frac{\mathrm{ADD}\times \mathrm{CSF}}{L}\;(R\;\leq \;0.01)$$

(11) $$\mathrm{ILCR}=\frac{1-\exp(-\mathrm{ADD}\times \mathrm{CSF})}{L}\;(R\;>\;0.01)$$

(12) TCR=∑ILCR

In general, if the TCR is >10^−4^, the carcinogenic risk is unacceptable; 1 × 10^−6^ < TCR < 1 × 10^−4^ is assumed to be acceptable or tolerable; and when the TCR < 10^−6^, it implies the carcinogenic risk can be ignored [37]. The values of the relevant parameters are shown in Tables S2 and S3.

#### 2.3.4. Monte Carlo Simulation

The MCS is a probabilistic and statistical-mathematical theory widely applied when selecting parameters for uncertainty analysis in risk assessment [38]. The exposure parameters, heavy metal concentrations, and the selection of health risk assessment models are the three main factors contributing to the uncertainty analysis of risk assessment [28]. In this study, MCS is employed to handle both uncertainty in heavy metal concentrations (Cw) and exposure parameters (such as exposure frequency (EF), exposure duration (ED) and ingestion rate (IR) of groundwater). The concentrations database of HMs fitted a lognormal distribution of an uncertain parameter (Table S4). The probability distribution functions of exposure factors applied in MCS are shown in Table S2. After ranking the health risk assessment results from low to high and counting the numbers falling in the predetermined frequency bins, the probability distribution plot was obtained to represent the output result. The number of trials of the random simulation is set to 10,000, and the confidence level is determined to be 95% to obtain the approximate solution of the risk assessment.

### 2.4. Statistical Analysis

Descriptive statistics and identification of sources were performed using SPSS 25, OriginPro 2021b and R software environment. Inverse distance weight interpolation was performed using ArcGIS 10.7 to analyze the spatial distribution of the concentrations of the HMs. The Monte Carlo simulation was performed with Crystal Ball 11.1.24 software.

## 3. Results and Discussion

### 3.1. Pollution Characterization of Heavy Metals

The descriptive statistics of HMs in the groundwater of Hainan Island (Table 1) showed that the mean values of Cr, Mn, Fe, Cu, Zn, Cd and Pb were 6.52, 175.39, 36.57, 1.57, 22.94, 2.17 and 0.05 μg/L, respectively. Except for Mn, all the mean values of the other six HMs did not exceed the grade III limits of the groundwater environmental quality standards (GB/T 14848-2017). Therefore, the environment protection department should focus on controlling the possible Mn pollution. Meanwhile, the maximum values of Mn and Fe were 18.26 and 17.08 times that of the mean values, respectively, suggesting that the aggregation trend of HMs at individual sampling sites is more obvious in the context of economic development, which may cause hazards to human health. In terms of the coefficient of variation (CV), except for Cr and Cu, the CVs of the other five HMs all surpassed 100%, with those of Mn and Fe being especially higher, indicating a large spatial distribution variation of these two kinds of elements. The ranking of skewness for all HMs is as follows: Fe > Mn > Cd > Zn > Cu > Pb > Cr, with Mn and Fe being more evident, which may be caused by anthropogenic effects.

In addition, the spatial distribution of the seven HMs (Figure S1) shows distinct regional differences. The high-value areas are mainly located in the western part of the island, and the low-value areas in the central-eastern part, which may be related to the local industrial layout. The western region has a high level of industrialization, and the burning of fossil fuels in the production and the emission of industrial waste easily cause the accumulation of HMs. The high-value areas of Cr and Pb are more widely distributed than the other HMs. The high-value areas of Fe, Cu, Zn and Cr show a punctiform distribution, possibly related to the high content at individual sampling sites. The high-value areas of Mn are mainly concentrated in the western and southern parts of the island, and the content distribution at some individual sampling sites is extremely high, which may be influenced by both human activities.

### 3.2. Pollution Level of Heavy Metals

**Figure 2:**
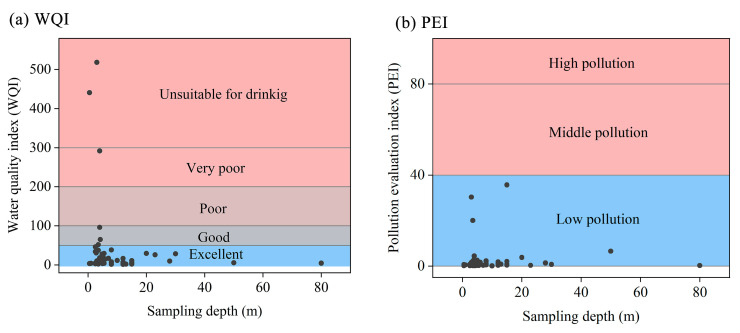
Classification of groundwater in Hainan Island based on (**a**) water quality index (WQI) and (**b**) pollution evaluation index (PEI).

The pollution levels by HMs are evaluated using the WQI and the PEI. The WQI results (Figure 2a) show that the WQI values range from 1.59 to 518.17 with a mean value of 35.60. For the mean values of WQI, the contribution of Fe, Cu, Mn, Zn, Cr, Pb and Cd reached 4.45, 0.06, 78.83, 0.84, 5.86, 9.76, 0.20%, respectively. The contributions of Mn and Pb are much higher than those of the other elements, which may be influenced by human activities. In addition, the WQI values at 95.00% of the sampling points was <100 and could be drunk directly, indicating that the quality of groundwater in Hainan Island is generally good, but it still needs further strengthening of the water quality risk management. However, it should be noted that there were two sampling points in the western industrial region with WQI values as high as 440.68 and 518.17, respectively, which was no longer suitable for drinking, implying the severe influence of industrial activities in terms of HM emissions. The PEI values range from 0.10 to 35.60 with a mean value of 2.41, indicating that all the sampling sites in the study area are of low pollution (Figure 2b). As for the mean PEI values, the contributions of Mn and Pb were 72.80 and 9.01%, respectively, with Mn and Pb being more striking, which further confirms the existence of the Mn and Pb pollution.

Meanwhile, the spatial distribution of WQI and PEI (Figure S2) shows that there is slight HM pollution in the western part of Hainan Island, followed by the eastern part, and almost no pollution in the central part, which is consistent with the fact that the central region of Hainan Island focuses on the development of ecological tourism with stricter control of the ecological environment, the eastern region focuses on the development of leisure agriculture with a relatively higher use of pesticides and fertilizers which may cause slight pollution of the soil and water, and the western region is dominated by heavy industries such as petroleum, chemical and mining with heavier emissions by traffic and industrial production. Therefore, further quantitative analysis of the pollution sources of HMs and identification of priority HMs for control are essential for the ecological protection and development of Hainan Island, especially in the western part.

### 3.3. Source Apportionment of Heavy Metals

**Figure 3:**
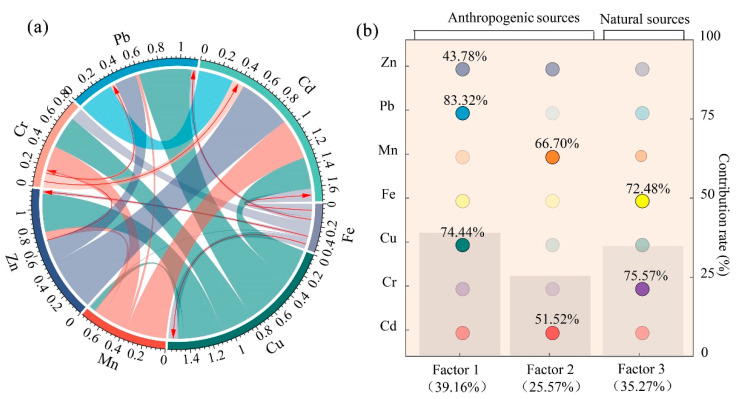
Source analysis of heavy metals in groundwater by combing (**a**) Pearson correlation analysis and (**b**) absolute principal component scores/multiple linear regression (APCS/MLR). (The width of each heavy metal is shown to denote the correlation coefficient. The red arrow denotes a negative correlation between two heavy metals. The histogram is used to represent the percentage of each factor. Different color gradients are used to indicate the proportions of each heavy metal to the different factors.).

The correlation analysis (CA) and the principal component analysis (PCA) were used to identify the sources of HMs in groundwater. The Pearson correlation coefficient results (*p* < 0.01) (Figure 3a) showed significant positive correlations (value of the correlation coefficient ranging between 0.35 and 0.49) between Zn and Cu, Pb and Cu, Cd and Mn, respectively, indicating that these HMs may have the same sources. Fe and Cr also presented a positive correlation (value of correlation coefficient is 0.12), while Fe and Cu, Fe and Zn, Fe and Cd, Fe and Pb presented negative correlations (value of correlation coefficient ranging between −0.12 and −0.04). This showed that regarding the pollutant source, Fe and Cr were likely different from other HMs.

The PCA is a tool that can effectively classify multiple HMs with similar sources. The maximum variance rotation method identified a total of three principal components (Table S5). The results of the KMO (0.503) and Bartlett tests (*p* < 0.001) demonstrate that the correlation among the variables is significant and the data of this study are suitable for the PCA. The three principal components, named as PC1, PC2 and PC3, respectively, cumulatively explained 65.60% of the total variance, and the contribution of PC1, PC2 and PC3 were 26.04, 21.22 and 18.34%, respectively. PC1 is heavily loaded with Cu, Zn and Pb; PC2 mainly includes Mn and Cd, with a slight loading of Zn; and PC3 is dominated by Fe and Cr.

Based on the PCA results, the APCS/MLR was used to further calculate the contribution rate of each pollution source to a single HM in the groundwater. The results show that the fitting coefficients (R^2^) between the predicted and measured concentrations of HMs range from 0.48 to 0.81, with ratios of the predicted and measured concentrations close to 1, indicating that the APCS/MLR model constructed is highly accurate and the calculation results are credible. Three factors, namely, Factor 1, Factor 2 and Factor 3, are identified by the APCS/MLR model, and their contribution rates are 39.16, 25.57 and 35.27%, respectively, as shown in Figure 3b.

Factor 1 is dominated by Cu (74.44%), Zn (43.78%) and Pb (83.32%). Cu and Zn are widely used in agricultural practices [39,40] in the forms of chemical fertilizers and pesticides which are used in large quantities every year in China’s agricultural production [41]. Hainan Island has a tropical climate, so diseases and insect pests are severe because of the high temperature and high humidity in the agricultural production environment. Meanwhile, the land is mostly red-yellow soil with low organic matter content, which leads to high frequency uses of pesticides and fertilizers. The statistical yearbooks show that the usage of pesticides and fertilizers increased by about 120 and 45 percent from 2005 to 2015 in Hainan Province, respectively [42], which should have influenced the related HM concentrations to some extent. Animal manure is another critical source of Zn and Cu. As a major agricultural province, animal husbandry has become the mainstay of local agricultural development in Hainan, and animal excretion may increase the content of HMs such as Zn and Cu. Pb may originate from pesticides and untreated domestic wastewater discharges [43]. Vehicle exhaust and atmospheric deposition are also important sources of Pb [44]. Hainan Island has convenient land and water transportation as a tourist destination and an agricultural and livestock production base. In addition, there are petrochemical plants, rubber factories, sugar factories and other enterprises. These factories need a lot of heavy vehicles to transport raw materials and finished products, and the HMs brought by the corrosion of vehicle parts, tire wear and gasoline leakage will accumulate. Thus, Factor 1 is mainly related to the agricultural activities, but still weakly influenced by traffic emissions.

Factor 2 is most related to Mn (66.70%) and Cd (51.52%). Usually, the variation of Mn content in groundwater is mainly controlled by factors such as geomorphology, the depositional environment of aquifers and hydraulic characteristics. However, several anomalous values of Mn were observed in the study area, revealing that it has also been influenced by human activities. Many previous studies have described that Mn and Cd were associated with industrial production [23,45]. The high-value areas of Mn and Cd (Figure S1) are mainly concentrated in the west of Hainan Island, sharing similar distribution characteristics with those enterprises covering the petroleum and chemical industry, electric and energy, mining and smelting, and paper-making and other fields, monitored highly by China in 2015 (Figure S3). On the one hand, the sewage from these enterprises will cause HM pollution of groundwater if not treated effectively. On the other, the mined metal ores and waste rock, piled and exposed on the surface, suffer from the weathering–leaching of meteoric water over a long time, and then form a large amount of acidic wastewater. The acidic wastewater carries many HMs infiltrated and spread into the groundwater which then cause pollution problems. Finally, the soil media on Hainan Island mainly consists of sandy sub clay and gravelly sub clay (Figure 1c), which have good permeability. Hence, this area may be easily affected by industrial effluent infiltration. Based on the analysis above, Factor 2 is mainly from the industrial activities.

Factor 3 is mainly associated with Cr (75.57%) and Fe (72.48%), which are mainly controlled by the geological background [46,47]. Cr and Fe are Fe-group elements with the same atomic composition and chemical valence state, which can substitute each other during mineral formation and are easily co-enriched in Fe-Cr minerals, resulting in a much higher content of Fe-Cr minerals in basal and ultramafic rocks than in other rock types. Combined with the spatial distributions of Cr and Fe (Figure S1), the high-value areas of these two kinds of elements are mainly distributed in the northern part of Hainan Island, where basaltic and metamorphic rocks are developed. The basalt is rich in Fe-Mg minerals such as pyroxene, and thus is enriched with high Cr and Fe contents. Therefore, Factor 3 can be interpreted as a natural source of HMs. The aquifer media of Hainan Island are shown in Figure 1d.

The above results of the APCS/MLR show three main sources of HMs in the groundwater, one is the mixed sources of agricultural activities and traffic emissions (39.16%), the other is industrial production (25.57%), and the third one is the geological background (35.27%). The mixed sources of agricultural activities and traffic emissions accounted for the highest percentage and should be the focus of HM control, especially the agricultural pollution. When the water environment in the study area is treated, effective measures can be implemented in combination with the specific sampling locations, types of HMs and possible pollution sources. For example, for the agricultural and traffic pollution represented by Factor 1, in addition to applying phytoremediation and biochar to the soil, the emission of automobile exhaust and the use of gasoline can also be remediated. For the industrial production represented by Factor 2, we should raise the entry threshold of polluting production capacity enterprises, strengthen the supervision of industrial production of key polluting enterprises, and promote the circular economy and clean production.

### 3.4. Probabilistic Health Risks Assessment

#### 3.4.1. Concentration-Oriented Health Risk Assessment

**Figure 4:**
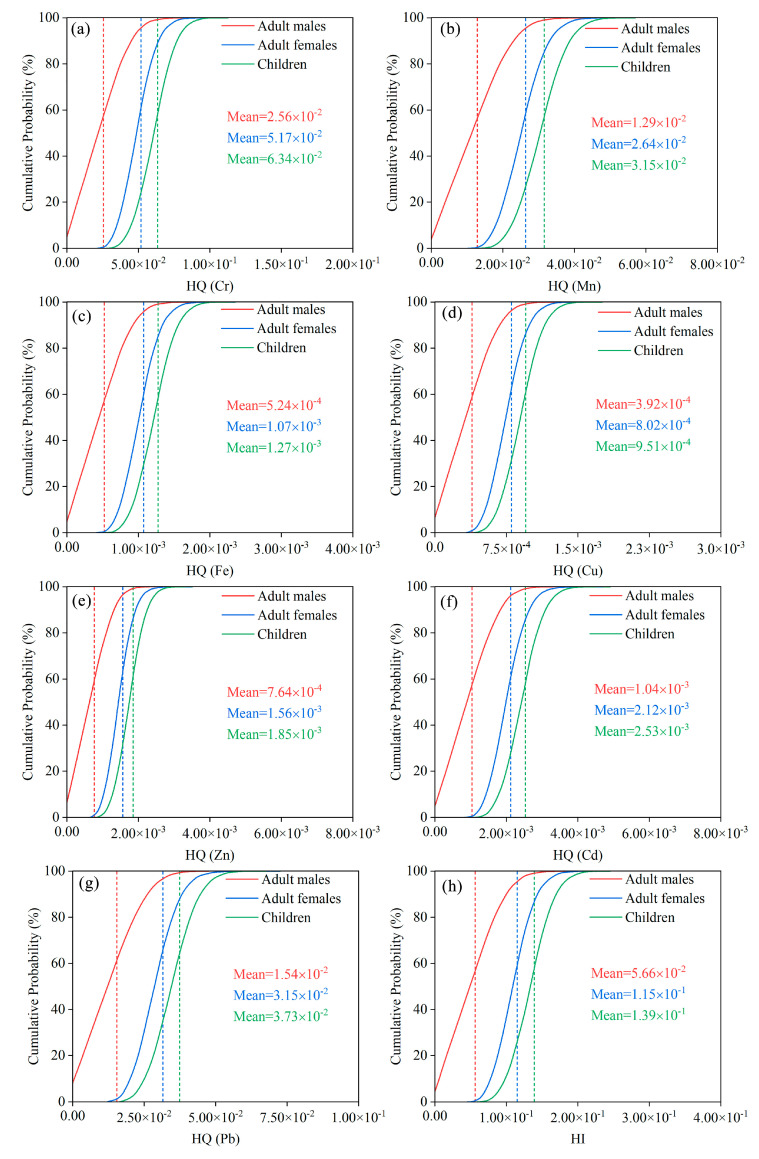
Probability distribution of hazard quotient (HQ) for (**a**) Cr, (**b**) Mn, (**c**) Fe, (**d**) Cu, (**e**) Zn, (**f**) Cd, (**g**) Pb, and (**h**) hazard index (HI) with the percentage of HQ and HI values surpassing 1, the guideline value (The blue, red, or green dashed vertical lines represent the mean values).

Based on the MCS, the health risks of the different populations (adult males, adult females, and children) exposed to the seven HMs in groundwater via two exposure pathways (ingestion and dermal contact) were assessed. In terms of non-carcinogenic risk, the average HQ value of the seven HMs was less than 1 (Table 2 and Figure 4), suggesting that no individual HM would cause the non-carcinogenic risk to local people. The basic trend of the mean HQ value for all groups is: Cr > Pb > Mn > Cd > Zn > Fe > Cu, with Cr and Pb posing higher non-carcinogenic risks to the population than the other HMs.

The HI values of all groups were less than 1, indicating that their non-carcinogenic risks are all negligible. It is worth noting that the mean HI value for children is 2.46 and 1.21 times higher than that of adult males and adult females, respectively, implying that the non-carcinogenic risk of children is significantly higher than that of adults, presumably for two reasons: first, the lower body weight and smaller skin surface area of children lead to their relatively higher average daily exposure dose. Second, children are more sensitive to external environmental influences during their growth and developmental stages. Previous studies have also concluded that children are more vulnerable to non-carcinogenic risks than adults [47,48], which is consistent with our study. In addition, taking children as a sensitive population, the contribution rates of Cr, Mn and Pb to the mean value of HI reached 45.67, 22.71 and 26.85%, respectively, and it is presumed that Cr, Mn and Pb should be the HMs that need priority control. Therefore, in the non-carcinogenic risk assessment of HMs in groundwater in this study area, we should focus on the health effects of Cr, Mn and Pb exposure on children.

**Figure 5:**
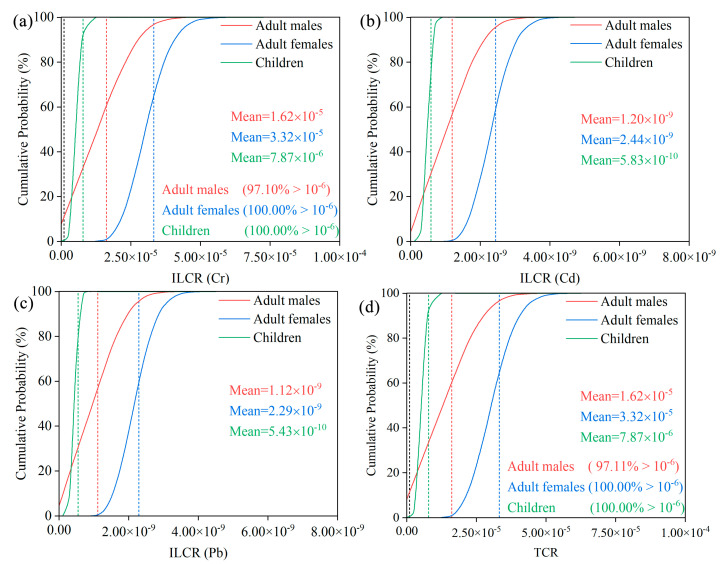
Probability distribution and the percentage of incremental lifetime cancer risk (ILCR) values surpassing 1.00 × 10^−6^ for (**a**) Cr, (**b**) Cd, (**c**) Pb, and (**d**) to the total cancer risk (TCR). (The red, blue, or green dashed vertical lines represent the mean values, whereas the black is the acceptable carcinogenic risk value of 1.00 × 10^−6^.).

As for carcinogenic risk, the mean values of ILCR for all groups decreased in the following order: Cr > Cd > Pb (Figure 5). Except for Cr, the mean ILCR values of Cd and Pb are much lower than the guidance value of 1.00 × 10^−6^ recommended by the USEPA, suggesting that the carcinogenic risk of Cd and Pb to the local people can be ignored. Meanwhile, although the mean TCR values for adult males, adult females and children are approximately 16, 33 and 8 times higher than the 1.00 × 10^−6^, respectively, they are all lower than the maximum acceptable risk value of 1.00 × 10^−4^, deducing that HMs in the study area have no significant long-term carcinogenic impact on the population.

In addition, among the three cancerogenic HMs, Cr has the highest carcinogenic risk, its contribution to the TCR is approximately 100.00%, whereas the contribution rates of Cd and Pb are the lowest, both less than 0.01%. Even at the fifth percentile, the mean values of ILCR of Cr for adult males, adult females and children were 1.62, 2.19 and 5.32 times higher than the 1.00 × 10^−6^ (Table S6), respectively, indicating that the population in the study area is likely to suffer from carcinogenic risk through drinking water or dermal exposure to Cr. The high carcinogenic risk of Cr can be explained by three reasons: first, the high content of Cr in the soil-forming parent rock of Hainan Island is influenced to some extent by the natural background values. Second, Hainan Island is located in the tropical region with high temperature and relatively higher drinking water consumption by the population. Third, the carcinogenic slope factor of Cr is relatively high, which increases the potential of Cr to cause health risks. Previous studies have also identified Cr as a major element contributing to the carcinogenic risk [49,50], and the health risks via the ingestion route are higher than the dermal contact route for both the carcinogenic risks and the non-carcinogenic risks (Table S7) [51,52], which are in agreement with this paper and all conclude that drinking water is the most dominant and direct pathway contributing to health risk. 

#### 3.4.2. Source-Oriented Health Risk Assessment

**Figure 6:**
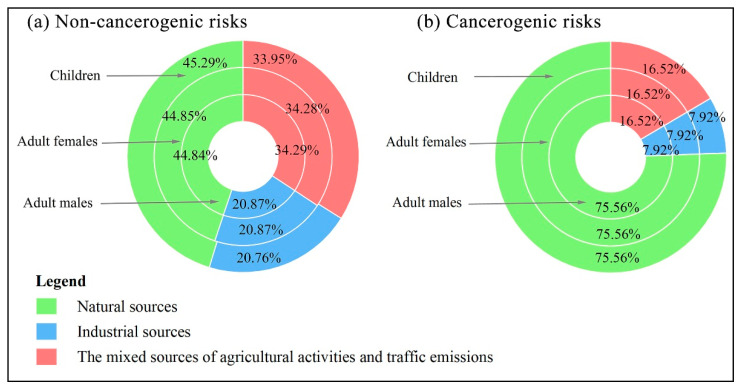
The contribution percentage of each pollution source to (**a**) the non-carcinogenic risk and (**b**) carcinogenic risk by heavy metals in groundwater for adult males, adult females, and children, respectively.

The result of APCS/MLR model was adopted to quantify the contribution of different sources to the health risks. The health risk values of individual HMs were multiplied by the contribution rates of identified sources to obtain the health risks caused by the different sources. For adult males, adult females and children, the contribution of different pollution sources to both the non-carcinogenic and the carcinogenic health risks shows the same trend, i.e., natural sources > the mixed sources of agricultural activities and traffic emissions > industrial activities (Figure 6). In the case of children, the contribution rates of the mixed sources of agricultural activities and traffic emissions, industrial activities and natural sources to the non-carcinogenic risk are 33.95, 20.76 and 45.29%, respectively, and the contribution rates to the carcinogenic risk are 16.52, 7.92 and 75.56%, respectively. Natural sources contribute the most to the health risks, manifesting that the geological background of Hainan Island influences the concentrations of HMs to a certain extent. In general, the contribution rate of soil parent material is higher than other sources. For example, HMs in soil are relatively high due to higher toxicity coefficients and higher Cr from soil material [27]. HMs in soil penetrate into groundwater reserves through rainfall and irrigation [36], leading to increased health risks of HMs in the groundwater.

Meanwhile, combined with the results of APCS/MLR, the contribution rate of natural sources to Cr is as high as 75.57%, indicating that Cr should be a key pollutant for prevention and control and special attention should be paid to its distribution in, and health risk to, the water. Industrial activities contribute the least to the health risks. As a national ecological civilization pilot zone and international free trade port, Hainan gives priority to ecological and environmental protection in the development process and vigorously develops a low-carbon economy, which ensures industrial activities having little impact on environmental pollution. The contribution of the mixed sources of agricultural activities and traffic emissions to the health risks is relatively low. Residents can reduce the output of agriculture and transportation to reduce the health risk of HMs in groundwater by reducing the use of chemical fertilizers and pesticides, increasing the recycling rate of mulch, and strengthening traffic management as much as possible. Finally, we should note that the high background values of HMs are very likely to be hazardous to human health. Therefore, it is necessary to study the pollution of HMs in groundwater in areas with high background concentration [23], which will enable a more comprehensive and objective assessment of the health risk of HMs in groundwater in the study area, and be more conducive to safeguarding the water environment of Hainan Island.

## 4. Conclusions

In this study, the pollution level, sources and health risks of HMs in the groundwater were assessed in Hainan Island. The results show that Mn, Fe and Pb are the most serious pollutants and contribute more to the groundwater pollution than the other HMs. By combining the APCS/MLR model and correlation analysis, in addition to the natural sources mentioned conventionally, the mixed sources of agricultural activities, traffic emissions and industrial activities are also identified as main pollution sources. The concentration-oriented and source-oriented health risks were assessed using the Monte Carlo simulation coupled with the APCS/MLR model, and the results revealed that the non-carcinogenic health risks for all populations are negligible, but the carcinogenic health risks all stay at a relatively high level. By sorting out the relationship between the HMs, pollution sources, and health risks, natural sources contribute the most to the health risks, and Cr is determined as the priority control HM. Overall, this study provides an effective approach for pollution control and water quality management policies in the groundwater. Furthermore, considering the natural sources contribute the most to health risks, the characteristics analysis of those areas with a higher background concentration of HMs should receive increased consideration in the future.

## Figures and Tables

**Table 1:** Statistical summary of concentrations of heavy metals (μg/L) in groundwater in Hainan Island.

	Min	Max	Mean	SD	CV/%	Skewness	Kurtosis	Guide value
Cr	1.69	16	6.52	3.12	47.8	0.93	1.1	50
Mn	0.01	3.2 × 10^3^	175	572	326	4.46	19.8	100
Fe	1.7	625	36.6	88.9	243	5.55	34.3	300
Cu	0.15	7.85	1.57	1.46	92.7	2.52	7.57	1.0 × 10^3^
Zn	1.66	137	22.9	28	122	2.58	6.83	1.0 × 10^3^
Cd	0	0.32	0.05	0.07	149	2.78	7.7	5
Pb	0.02	12.9	2.17	3.59	165	1.82	2.04	100

**Table 2:** Summary of statistics for non-carcinogenic and carcinogenic health risk results via Monte Carlo simulation.

Risk	Metal	Mean (Median)			SD			95% CI		
		Adult Males	Adult Females	Children	Adult Males	Adult Females	Children	Adult Males	Adult Females	Children
HQ	Cr	2.56 × 10^−2^ (2.45 × 10^−2^)	5.17 × 10^−2^ (5.09 × 10^−2^)	6.34 × 10^−2^ (6.32 × 10^−2^)	1.58 × 10^−2^	1.13 × 10^−2^	1.23 × 10^−2^	(2.53 × 10^−2^, 2.59 × 10^−2^)	(5.15 × 10^−2^, 5.19 × 10^−2^)	(6.31 × 10^−2^, 6.36 × 10^−2^)
	Mn	1.29 × 10^−2^ (1.24 × 10^−2^)	2.64 × 10^−2^ (2.60 × 10^−2^)	3.15 × 10^−2^ (3.14 × 10^−2^)	8.03 × 10^−3^	5.87 × 10^−3^	6.31 × 10^−3^	(1.28 × 10^−2^, 1.31 × 10^−2^)	(2.63 × 10^−2^, 2.65 × 10^−2^)	(3.14 × 10^−2^, 3.16 × 10^−2^)
	Fe	5.24 × 10^−4^ (5.01 × 10^−4^)	1.07 × 10^−3^ (1.06 × 10^−3^)	1.27 × 10^−3^ (1.27 × 10^−3^)	3.26 × 10^−4^	2.40 × 10^−4^	2.57 × 10^−4^	(5.18 × 10^−4^, 5.31 × 10^−4^)	(1.07 × 10^−3^, 1.08 × 10^−3^)	(1.27 × 10^−3^, 1.28 × 10^−3^)
	Cu	3.92 × 10^−4^ (3.74 × 10^−4^)	8.02 × 10^−4^ (7.90 × 10^−4^)	9.51 × 10^−4^ (9.47 × 10^−4^)	2.44 × 10^−4^	1.80 × 10^−4^	1.92 × 10^−4^	(3.87 × 10^−4^, 3.97 × 10^−4^)	(7.98 × 10^−4^, 8.05 × 10^−4^)	(9.47 × 10^−4^, 9.55 × 10^−4^)
	Zn	7.64 × 10^−4^ (7.29 × 10^−4^)	1.56 × 10^−3^ (1.54 × 10^−3^)	1.85 × 10^−3^ (1.84 × 10^−3^)	4.75 × 10^−4^	3.50 × 10^−4^	3.75 × 10^−4^	(7.55 × 10^−4^, 7.73 × 10^−4^)	(1.56 × 10^−3^, 1.57 × 10^−3^)	(1.84 × 10^−3^, 1.86 × 10^−3^)
	Cd	1.04 × 10^−3^ (9.94 × 10^−4^)	2.12 × 10^−3^ (2.08 × 10^−3^)	2.53 × 10^−3^ (2.52 × 10^−3^)	6.45 × 10^−4^	4.71 × 10^−4^	5.06 × 10^−4^	(1.03 × 10^−3^, 1.05 × 10^−3^)	(2.11 × 10^−3^, 2.13 × 10^−3^)	(2.52 × 10^−3^, 2.54 × 10^−3^)
	Pb	1.54 × 10^−2^ (1.47 × 10^−2^)	3.15 × 10^−2^ (3.10 × 10^−2^)	3.73 × 10^−2^ (3.71 × 10^−2^)	9.56 × 10^−3^	7.06 × 10^−3^	7.55 × 10^−3^	(1.52 × 10^−2^, 1.56 × 10^−2^)	(3.13 × 10^−2^, 3.16 × 10^−2^)	(3.71 × 10^−2^, 3.74 × 10^−2^)
HI	Total	5.66 × 10^−2^ (5.42 × 10^−2^)	1.15 × 10^−1^ (1.13 × 10^−1^)	1.39 × 10^−1^ (1.38 × 10^−1^)	3.51 × 10^−2^	2.54 × 10^−2^	2.75 × 10^−2^	(5.59 × 10^−2^, 5.73 × 10^−2^)	(1.15 × 10^−1^, 1.16 × 10^−1^)	(1.38 × 10^−1^, 1.39 × 10^−1^)
ILCR	Cr	1.62 × 10^−5^ (1.55 × 10^−5^)	3.32 × 10^−5^ (3.27 × 10^−5^)	7.87 × 10^−6^ (7.83 × 10^−6^)	1.01 × 10^−5^	7.45 × 10^−6^	1.59 × 10^−6^	(1.60 × 10^−5^, 1.64 × 10^−5^)	(3.31 × 10^−5^, 3.34 × 10^−5^)	(7.84 × 10^−6^, 7.90 × 10^−6^)
	Cd	1.20 × 10^−9^ (1.14 × 10^−9^)	2.44 × 10^−9^ (2.41 × 10^−9^)	5.83 × 10^−10^ (5.81 × 10^−10^)	7.44 × 10^−10^	5.44 × 10^−10^	1.17 × 10^−10^	(1.18 × 10^−9^, 1.21 × 10^−9^)	(2.43 × 10^−9^, 2.45 × 10^−9^)	(5.81 × 10^−10^, 5.85 × 10^−10^)
	Pb	1.12 × 10^−9^ (1.07 × 10^−9^)	2.29 × 10^−9^ (2.26 × 10^−9^)	5.43 × 10^−10^ (5.40 × 10^−10^)	6.97 × 10^−10^	5.14 × 10^−10^	1.10 × 10^−10^	(1.11 × 10^−9^, 1.13 × 10^−9^)	(2.28 × 10^−9^, 2.30 × 10^−9^)	(5.41 × 10^−10^, 5.45 × 10^−10^)
TCR	Total	1.62 × 10^−5^ (1.55 × 10^−5^)	3.32 × 10^−5^ (3.27 × 10^−5^)	7.87 × 10^−6^ (7.83 × 10^−6^)	1.01 × 10^−5^	7.45 × 10^−6^	1.59 × 10^−6^	(1.60 × 10^−5^, 1.64 × 10^−5^)	(3.31 × 10^−5^, 3.34 × 10^−5^)	(7.84 × 10^−6^, 7.90 × 10^−6^)

Abbreviations: HQ, hazard quotient of each heavy metal; HI, hazard index posed by multiple HMs; CR, cancer risk of each heavy metal; TCR, total cancer risks posed by multiple heavy metals; SD, standard deviation; CI, confidential int.

## Data Availability

No new data were created or analyzed in this study. Data sharing is not applicable to this article.

## Supplementary Materials

The following supporting information can be downloaded at: https://www.mdpi.com/article/10.3390/ijerph19137827/s1, Table S1: Parameters used for calculation of water quality index (WQI); Table S2: Exposure parameters used for health risk assessment calculations in this study; Table S3: Values of reference dose (RfD) and carcinogenic slope factor (CSF) in different pathways; Table S4: Uncertain concentration (μg/L) of heavy metals (HMs) in groundwater of Hainan Island; Table S5: Principal component analysis of heavy metals in groundwater of Hainan Island; Table S6: Summary statistics for 5th, 25th, 50th, 75th and 95th percentile non-carcinogenic and carcinogenic health risks based on Monte Carlo simulation using Crystal Ball (vs. 11.1.2.4); Table S7: Non-carcinogenic and carcinogenic health risks via different pathways; Figure S1: Spatial distribution of the concentrations (μg/L) of groundwater heavy metals in study area; Figure S2: Spatial distribution of (a) the water quality index (WQI) and (b) pollution evaluation index (PEI); Figure S3: The country’s key monitoring enterprises in Hainan province in 2015. References [22,28,29,31,35,37,52,53,54,55,56,57,58,59] are cited in the supplementary materials.
