# Supplementary material for: Health Risk Assessment of Heavy Metals in Groundwater of Hainan Island Using the Monte Carlo Simulation Coupled with the APCS/MLR Model
Source: Int J Environ Res Public Health. 2022 Jun 26;19(13):7827. doi: 10.3390/ijerph19137827 (PMC9266011; doi:10.3390/ijerph19137827)
Supplement: Supplementary file 1 [file ijerph-19-07827-s001.zip › ijerph-1684990-supplementary.pdf]

Supplementary Material

Tables and Figures list

Table S1. Parameters used for calculation of water quality index (WQI).

Table S2. Exposure parameters used for health risk assessment calculations in this study.

Table S3. Values of reference dose (RfD) and carcinogenic slope factor (CSF) in different pathways.

Table S4. Uncertain concentration (ug/L) of heavy metals (HMs) in groundwater of Hainan Island.

Table S5. Principal component analysis of heavy metals in groundwater of Hainan Island.

Table S6. Summary statistics for 5th, 25th, 50th, 75th and 95th percentile non-carcinogenic and carcinogenic health risk based on Monte Carlo simulation using Crystal Ball (vs. 11.1.2.4).

Table S7 Non-carcinogenic and carcinogenic health risk via different pathways.

Figure S1. Spatial distribution of the concentrations (ug/L) of groundwater heavy metals in study area.

Figure S2. Spatial distribution of (a) the water quality index (WQI) and (b) pollution evaluation index (PEI).

Figure S3. The country's key monitoring enterprises in Hainan province in 2015.

Table S1. Parameters used for calculation of water quality index (WQI).

Parameters	wi	Wi	Si (mg/L)
Cr	5.00 [1]	0.16	0.05
Mn	5.00 [1]	0.16	0.10
Fe	4.00 [2]	0.13	0.30
Cu	4.00 [2]	0.13	1.00
Zn	4.00 [1]	0.13	1.00
Cd	5.00 [1]	0.16	0.01
Pb	5.00 [1]	0.16	0.01
Total	32.00	1.00	

Table S2. Exposure parameters used for health risk assessment calculations in this study.

Parameters	Unit	Probabilistic Distribution	Children	Adult females	Adult males	References
C_w	mg L	Normal	Measured	Measured	Measured	This study
Intake rate (IR)	L/day	Normal	50th : 0.6, 95th : 1.3	50th : 1.4, 95th : 3.4	50th : 1.6, 95th : 4.0	[3,4]
Exposure frequency (EF)	day/year	Triangular	350 (180, 365)	350 (180, 365)	350 (180, 365)	[5]
Exposure duration (ED)	year	Uniform	(0, 6)	(0, 30)	(0, 30)	[6,7]
Average body weight (BW)	kg	Lognormal	(19.6, 1.96)	(57.59, 8.03)	(67.55, 8.72)	[3,4]
Average time of exposure (AT)	day	point	365 × ED (non-carcinogenic) 365 × 70 (carcinogenic)			[5,6]
Exposed skin area (SA)	cm^2	Normal	50th : 8000, 95th : 9500	50th : 15000, 95th : 20000	50th : 17000, 95th : 20000	[3,4]
Skin permeability coefficient (PC)	cm/h	point	0.002 (Cr), 0.0001 (Mn), 0.001 (Fe and Cd), 0.0006 (Zn and Cu), 0.00004 (Pb)			[8-10]
Exposure time (ET)	h/day	Triangular	0.20 (0.13, 0.33)	0.20 (0.13, 0.33)	0.20 (0.13, 0.33)	[11,12]
Unit conversion factor (CF)	L/cm^3	point	0.001	0.001	0.001	[13]
Average Life（L）	a	Fixed value	70	70	70	[10]

Notes: a1 and a2 in Log-Normal and Normal (a1, a2) defines the average value and the standard deviation for logarithmic normal and normal distribution; b1 and b2 in Uniform (b1, b2) defines the minimum and the maximum for uniform distribution; c1, c2 and c3 in in Triangular c1(c2, c3) defines the most likely value, the minimum and the maximum for triangular distribution.

Table S3. Values of reference dose (RfD) and carcinogenic slope factor (CSF) in different pathways.

Parameters	Cr	Mn	Fe	Cu	Zn	Cd	Pb
RfD_{oral}	3.00×10^{-3} [a]	1.40×10^{-1} [b]	7.00×10^{-1} [a]	4.00×10^{-2} [a]	3.00×10^{-1} [a]	5.00×10^{-4} [a]	1.40×10^{-3} [a]
RfD_{dermal}	7.50×10^{-5} [a]	8.00×10^{-4} [b]	1.40×10^{-1} [a]	8.00×10^{-3} [a]	6.00×10^{-2} [a]	2.50×10^{-5} [a]	4.20×10^{-4} [a]
CSF_{oral}	4.10×10^{1} [b]	–	–	–	–	3.8×10^{-1} [b]	8.50×10^{-3} [a]
CSF_{dermal}	5.00×10^{-1} [b]	–	–	–	–	6.10 [b]	n/a

Note: n/a not available. a.[14]; b. [10]

Table S4. Uncertain concentration (ug/L) of heavy metals (HMs) in groundwater of Hainan Island.

Parameters	Probabilistic distribution	Parameters（mean, SD）	Reference
Cr	Lognormal	(6.52, 3.12)	Metal specific
Mn	Lognormal	(175.39, 572.48)	Metal specific
Fe	Lognormal	(36.57, 88.90)	Metal specific
Cu	Lognormal	(1.57, 1.46)	Metal specific
Zn	Lognormal	(22.94, 27.99)	Metal specific
Cd	Lognormal	(0.05, 0.07)	Metal specific
Pb	Lognormal	(2.17, 3.59)	Metal specific

Table S5. Principal component analysis of heavy metals in groundwater of Hainan Island.

Component	Initial eigenvalues			Extraction sums of squared loadings			Rotation sums of squared loadings		
	Total	% of Variance	Cumulative %	Total	% of Variance	Cumulative %	Total	% of Variance	Cumulative %
1	2.19	31.24	31.24	2.19	31.24	31.24	1.82	26.04	26.04
2	1.28	18.34	49.58	1.28	18.34	49.58	1.49	21.22	47.26
3	1.12	16.02	65.60	1.12	16.02	65.60	1.28	18.34	65.60
4	0.95	13.53	79.13	—	—	—	—	—	—
5	0.78	11.12	90.26	—	—	—	—	—	—
6	0.35	5.04	95.29	—	—	—	—	—	—
7	0.33	4.71	100.00	—	—	—	—	—	—

Note: "—" characteristic root component is not less than 1 and rotation.

Table S6. Summary statistics for 5th, 25th, 50th, 75th and 95th percentile non-carcinogenic and carcinogenic health risk based on Monte Carlo simulation using Crystal Ball (vs. 11.1.2.4).

Risk	Metal	Adult males					Adult females					Children				
		5th	25th	50th	75th	95th	5th	25th	50th	75th	95th	5th	25th	50th	75th	95th
HQ	Cr	2.56×10^{-3}	1.25×10^{-2}	2.45×10^{-2}	3.72×10^{-2}	5.27×10^{-2}	3.43×10^{-2}	4.36×10^{-2}	5.09×10^{-2}	5.90×10^{-2}	7.12×10^{-2}	4.35×10^{-2}	5.47×10^{-2}	6.32×10^{-2}	7.16×10^{-2}	8.41×10^{-2}
	Mn	1.30×10^{-3}	6.29×10^{-3}	1.24×10^{-2}	1.88×10^{-2}	2.67×10^{-2}	1.74×10^{-2}	2.22×10^{-2}	2.60×10^{-2}	3.01×10^{-2}	3.65×10^{-2}	2.14×10^{-2}	2.71×10^{-2}	3.14×10^{-2}	3.56×10^{-2}	4.23×10^{-2}
	Fe	5.26×10^{-5}	2.55×10^{-4}	5.01×10^{-4}	7.61×10^{-4}	1.09×10^{-3}	7.07×10^{-4}	9.00×10^{-4}	1.06×10^{-3}	1.23×10^{-3}	1.49×10^{-3}	8.62×10^{-4}	1.09×10^{-3}	1.27×10^{-3}	1.44×10^{-3}	1.71×10^{-3}
	Cu	3.93×10^{-5}	1.91×10^{-4}	3.74×10^{-4}	5.69×10^{-4}	8.12×10^{-4}	5.29×10^{-4}	6.73×10^{-4}	7.90×10^{-4}	9.17×10^{-4}	1.11×10^{-3}	6.44×10^{-4}	8.15×10^{-4}	9.47×10^{-4}	1.08×10^{-3}	1.28×10^{-3}
	Zn	7.65×10^{-5}	3.71×10^{-4}	7.29×10^{-4}	1.11×10^{-3}	1.58×10^{-3}	1.03×10^{-3}	1.31×10^{-3}	1.54×10^{-3}	1.79×10^{-3}	2.17×10^{-3}	1.25×10^{-3}	1.59×10^{-3}	1.84×10^{-3}	2.10×10^{-3}	2.49×10^{-3}
	Cd	1.04×10^{-4}	5.05×10^{-4}	9.94×10^{-4}	1.51×10^{-3}	2.14×10^{-3}	1.40×10^{-3}	1.78×10^{-3}	2.08×10^{-3}	2.42×10^{-3}	2.93×10^{-3}	1.72×10^{-3}	2.17×10^{-3}	2.52×10^{-3}	2.86×10^{-3}	3.40×10^{-3}
	Pb	1.54×10^{-3}	7.48×10^{-3}	1.47×10^{-2}	2.23×10^{-2}	3.19×10^{-2}	2.07×10^{-2}	2.64×10^{-2}	3.10×10^{-2}	3.60×10^{-2}	4.37×10^{-2}	2.52×10^{-2}	3.19×10^{-2}	3.71×10^{-2}	4.22×10^{-2}	5.02×10^{-2}
HI	Total	5.65×10^{-3}	2.76×10^{-2}	5.42×10^{-2}	8.23×10^{-2}	1.17×10^{-1}	7.61×10^{-2}	9.68×10^{-2}	$1.13\times1^{0-1}$	1.31×10^{-1}	1.59×10^{-1}	9.46×10^{-2}	1.19×10^{-1}	1.38×10^{-1}	1.57×10^{-1}	1.86×10^{-1}
ILCR	Cr	1.62×10^{-6}	7.89×10^{-6}	1.55×10^{-5}	2.36×10^{-5}	3.36×10^{-5}	2.19×10^{-5}	2.79×10^{-5}	3.27×10^{-5}	3.80×10^{-5}	4.61×10^{-5}	5.32×10^{-6}	6.74×10^{-6}	7.83×10^{-6}	8.90×10^{-6}	1.06×10^{-5}
	Cd	1.20×10^{-10}	5.83×10^{-10}	1.14×10^{-9}	1.74×10^{-9}	2.48×10^{-9}	1.61×10^{-9}	2.05×10^{-9}	2.41×10^{-9}	2.79×10^{-9}	3.38×10^{-9}	3.96×10^{-10}	5.00×10^{-10}	5.81×10^{-10}	6.59×10^{-10}	7.83×10^{-10}
	Pb	1.12×10^{-10}	5.44×10^{-10}	1.07×10^{-9}	1.63×10^{-9}	2.32×10^{-9}	1.51×10^{-9}	1.92×10^{-9}	2.26×10^{-9}	2.62×10^{-9}	3.18×10^{-9}	3.67×10^{-10}	4.65×10^{-10}	5.40×10^{-10}	6.14×10^{-10}	7.31×10^{-10}
TCR	Total	1.62×10^{-6}	7.89×10^{-6}	1.55×10^{-5}	2.36×10^{-5}	3.36×10^{-5}	2.19×10^{-5}	2.79×10^{-5}	3.27×10^{-5}	3.80×10^{-5}	4.61×10^{-5}	5.32×10^{-6}	6.74×10^{-6}	7.83×10^{-6}	8.90×10^{-6}	1.06×10^{-5}

Table S7. Non-carcinogenic and carcinogenic health risk via different pathways.

Risk	Metal	Adult males		Adult males		Children	
		Ingestion	Dermal contact	Ingestion	Dermal contact	Ingestion	Dermal contact
Non-carcinogenic risk	Cr	2.16×10^{-2}	4.02×10^{-3}	4.41×10^{-2}	7.56×10^{-3}	5.22×10^{-2}	1.11×10^{-2}
	Mn	1.24×10^{-2}	5.06×10^{-4}	2.54×10-^{2}	9.53×10^{-4}	3.01×10^{-2}	1.41×10^{-3}
	Fe	5.18×10^{-4}	6.03×10^{-6}	1.06×10^{-3}	1.14×10^{-5}	1.26×10^{-3}	1.67×10^{-5}
	Cu	3.89×10^{-4}	2.72×10^{-6}	7.97×10^{-4}	5.12×10^{-6}	9.43×10^{-4}	7.55×10^{-6}
	Zn	7.59×10^{-4}	5.30×10^{-6}	1.55×10^{-3}	9.97×10^{-6}	1.84×10^{-3}	1.47×10^{-5}
	Cd	9.92×10^{-4}	4.62×10^{-5}	2.03×10^{-3}	8.69×10^{-5}	2.40×10^{-3}	1.28×10^{-4}
	Pb	1.54×10^{-2}	4.77×10^{-6}	3.15×10^{-2}	8.98×10^{-6}	10	1.32×10^{-5}
	HI	5.20×10^{-2}	4.59×10^{-3}	1.06×10^{-1}	8.63×10^{-3}	1.26×10^{-1}	1.27×10^{-2}
Carcinogenic risk	Cr	1.62×10^{-5}	9.22×10^{-10}	3.32×10^{-5}	1.73×10^{-9}	7.87×10^{-6}	5.12×10^{-10}
	Cd	1.15×10^{-9}	4.31×10^{-11}	2.36×10^{-9}	8.12×10^{-11}	5.59×10^{-10}	2.39×10^{-11}
	Pb	1.12×10^{-9}	-	2.29×10^{-9}	-	5.43×10^{-10}	-
	TCR	1.62×10^{-5}	9.65×10^{-10}	3.32×10^{-5}	1.82×10^{-9}	7.87×10^{-6}	5.36×10^{-10}

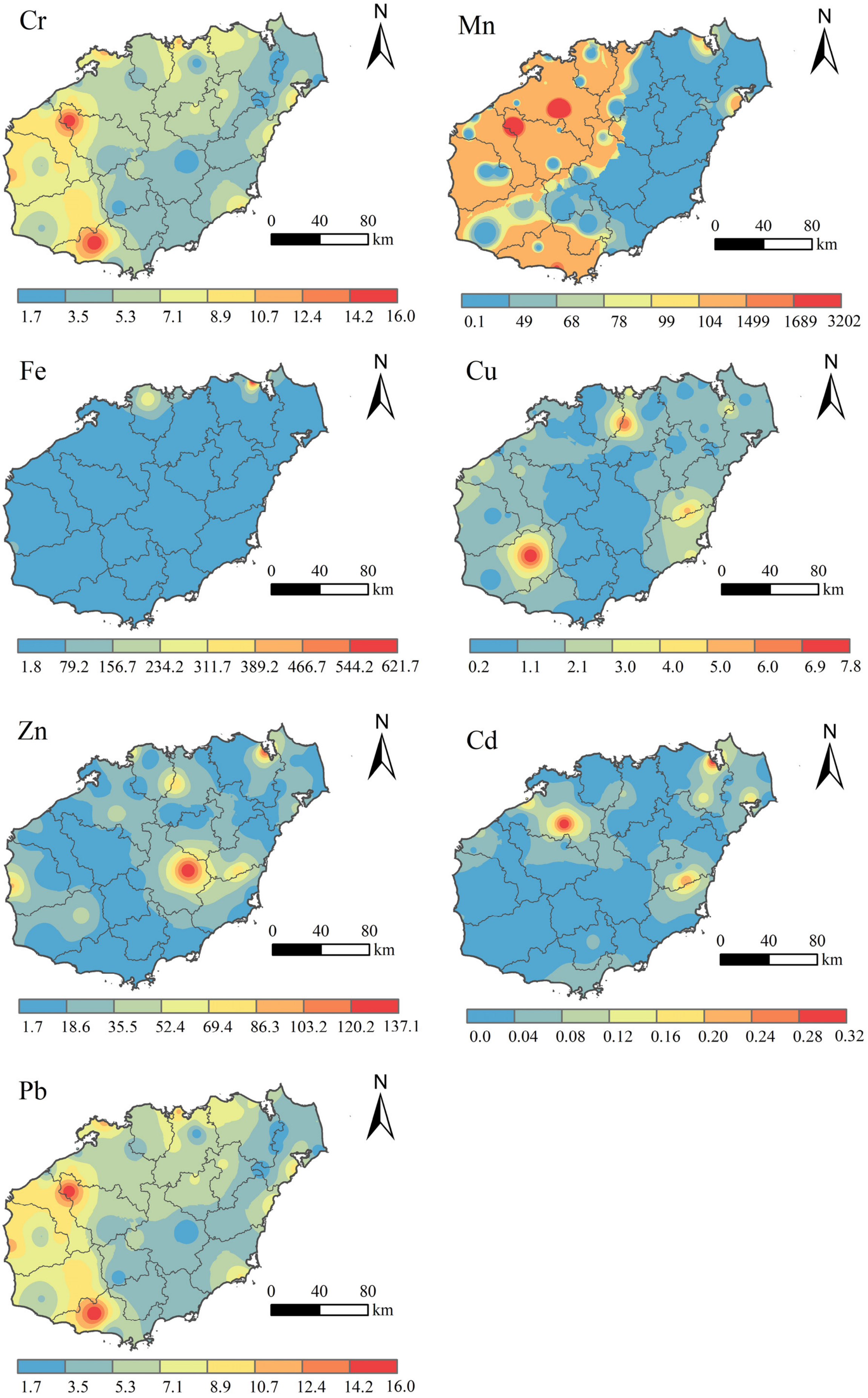

Figure S1. Spatial distribution of the concentrations (ug/L) of heavy metals in groundwater in study area.

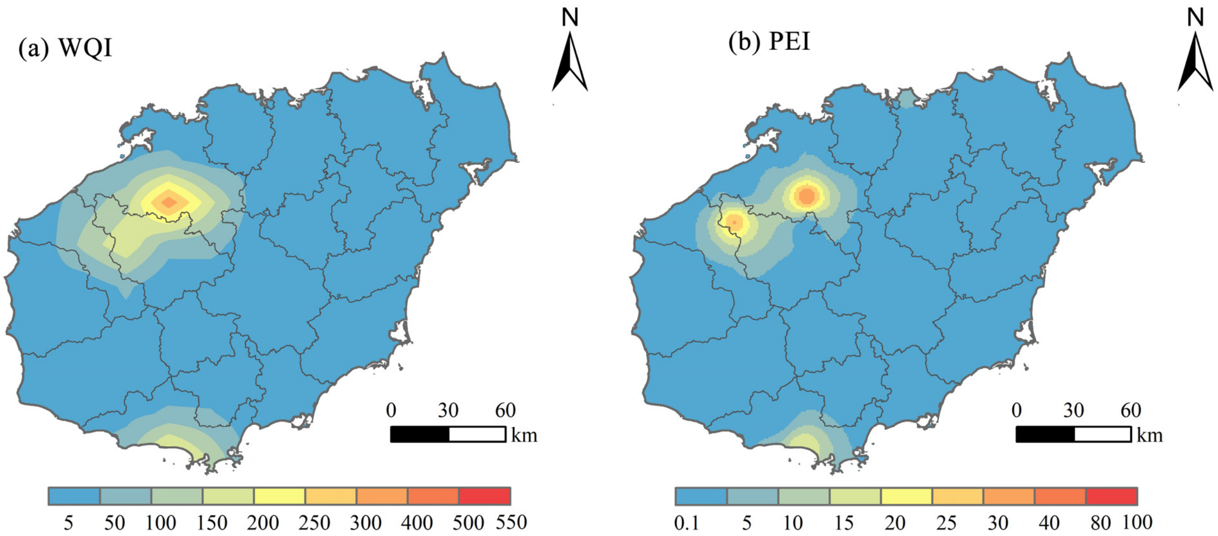

Figure S2. Spatial distribution of (**a**) the water quality index (WQI) and (**b**) pollution evaluation index (PEI).

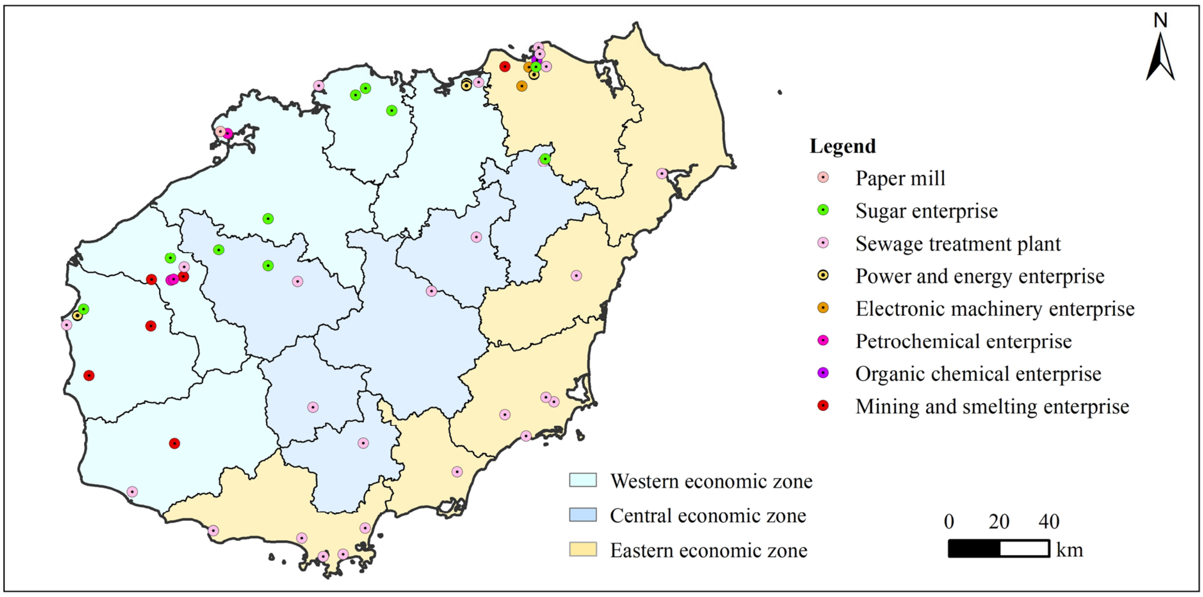

Figure S3. The country's key monitoring enterprises in Hainan province in 2015.

References

1. Githaiga, K.B.; Njuguna, S.M.; Gituru, R.W.; Yan, X. Water quality assessment, multivariate analysis and human health risks of heavy metals in eight major lakes in Kenya. *J. Environ. Manag.* **2021**, *297*, 113410. https://doi.org/10.1016/j.jenvman.2021.113410.
2. Egbueri, J.C. Heavy metals pollution source identification and probabilistic health Risk Assessment of Shallow Groundwater in Onitsha, Nigeria. *Anal. Lett.* **2020**, 53, 1620–1638. https://doi.org/10.1080/00032719.2020.1712606.
3. MEP. *Highlights of the Chinese Exposure Factors Handbook (Adults)*; China Environmental Science Press: Beijing, China, 2014. (In Chinese)
4. MEP. *Highlights of the Chinese Exposure Factors Handbook (Children)*; China Environmental Science Press: Beijing, China, 2016. (In Chinese)
5. Huang, J.; Wu, Y.; Sun, J.; Li, X.; Geng, X.; Zhao, M.; Sun, T.; Fan, Z. Health risk assessment of heavy metal(loid)s in park soils of the largest megacity in China by using Monte Carlo simulation coupled with Positive matrix factorization model. *J. Hazard. Mater.* **2021**, 415, 125629. https://doi.org/10.1016/j.jhazmat.2021.125629
6. Ngo, H.T.T.; Watchalayann, P.; Nguyen, D.B.; Doan, H.N.; Liang, L. Environmental health risk assessment of heavy metal exposure among children living in an informal e-waste processing village in Viet Na. *Sci. Total Environ.* **2021**, *763*, 142982. https://doi.org/10.1016/j.scitotenv.2020.142982
7. Zeng, G.; Liang, J.; Guo, S.; Shi, L.; Xiang, L.; Li, X.; Du, C. Spatial analysis of human health risk associated with ingesting manganese in Huangxing Town, Middle China. *Chemosphere* **2009**, 77, 368–375. https://doi.org/10.1016/j.chemosphere.2009.07.020
8. USEPA. Risk assessment guidance for superfund. In *Part A: Human Health Evaluation Manual; Part E, Supplemental Guidance for Dermal Risk Assessment, Vol. 1*; U.S. Environment Protection Agency: Washington, DC, USA, 2011.
9. Duan, X. Research Methods of Exposure Factors and Its Application in Environmental Health Risk Assessment; Science Press: Beijing, China, 2012.
10. Jiang, C.; Zhao, Q.; Zheng, L.; Chen, X.; Li, C.; Ren, M. Distribution, source and health risk assessment based on the Monte Carlo method of heavy metals in shallow groundwater in an area affected by mining activities, China. *Ecotox. Environ. Safe.* **2021**, *224*, 112679. https://doi.org/10.1016/j.ecoenv.2021.112679.
11. Saha, N.; Rahman, M.S.; Ahmed, M.B.; Zhou, J.L.; Ngo, H.H.; Guo, W. Industrial metal pollution in water and probabilistic assessment of human health risk. *J. Environ. Manag.* **2017**, *185*, 70–78. https://doi.org/10.1016/j.jenvman.2016.10.023.
12. USEPA. Quantitative Uncertainty Analysis of Superfund Residential Risk Pathway Models for Soil and Groundwater: White Paper; US Environmental Protection Agency: Washington, DC, USA, 1996.
13. Nilkarnjanakul, W.; Watchalayann, P.; Chotpantarat, S. Spatial distribution and health risk assessment of As and Pb contamination in the groundwater of Rayong Province, Thailand. *Environ. Res.* **2022**, *204*, 111838. https://doi.org/10.1016/j.envres.2021.111838.
14. Panda, G.; Pobi, K.K.; Gangopadhyay, S.; Gope, M.; Rai, A.K.; Nayek, S. Contamination level, source identification and health risk evaluation of potentially toxic elements (PTEs) in groundwater of an industrial city in eastern India. *Environ. Geochem. Health* **2021**, 1–25. https://doi.org/10.1007/s10653-021-01071-1.
